# Autophagy Inhibition Reduces Irradiation-Induced Subcortical White Matter Injury Not by Reducing Inflammation, but by Increasing Mitochondrial Fusion and Inhibiting Mitochondrial Fission

**DOI:** 10.1007/s12035-021-02653-x

**Published:** 2021-12-28

**Authors:** Yafeng Wang, Yiran Xu, Kai Zhou, Shan Zhang, Yong Wang, Tao Li, Cuicui Xie, Xiaoli Zhang, Juan Song, Xiaoyang Wang, Changlian Zhu

**Affiliations:** 1 Henan Provincial Key Laboratory of Children’s Genetics and Metabolic Diseases, Children’s Hospital Affiliated To Zhengzhou University, Henan Children’s Hospital, Zhengzhou Children’s Hospital, Zhengzhou, 450018 China; 2 Henan Key Laboratory of Child Brain Injury and Henan Pediatric Clinical Research Center, Institute of Neuroscience and Third Affiliated Hospital of Zhengzhou University, Zhengzhou, 450052 China; 3 Center for Brain Repair and Rehabilitation, Institute of Neuroscience and Physiology, Sahlgrenska Academy, University of Gothenburg, 40530 Göteborg, Sweden; 4 Department of Women’s and Children’s Health, Karolinska Institute, 17176 Stockholm, Sweden; 5 Centre for Perinatal Medicine and Health, Institute of Clinical Sciences, Sahlgrenska Academy, Gothenburg University, 40530 Gothenburg, Sweden

**Keywords:** Autophagy, Microglia, Astrocyte, White matter injury, Mitochondria, Fusion and fission

## Abstract

Radiotherapy is an effective tool in the treatment of malignant brain tumors, but irradiation-induced late-onset toxicity remains a major problem. The purpose of this study was to investigate if genetic inhibition of autophagy has an impact on subcortical white matter development in the juvenile mouse brain after irradiation. Ten-day-old selective neural *Atg7* knockout (KO) mice and wild-type (WT) littermates were subjected to a single 6-Gy dose of whole-brain irradiation and evaluated at 5 days after irradiation. Neural *Atg7* deficiency partially prevented myelin disruption compared to the WT mice after irradiation, as indicated by myelin basic protein staining. Irradiation induced oligodendrocyte progenitor cell loss in the subcortical white matter, and *Atg7* deficiency partly prevented this. There was no significant change between the KO and WT mice in the number of microglia and astrocytes in the subcortical white matter after irradiation. Transcriptome analysis showed that the GO mitochondrial gene expression pathway was significantly enriched in the differentially expressed genes between the KO and WT group after irradiation. Compared with WT mice, expression of the mitochondrial fusion protein OPA1 and phosphorylation of the mitochondrial fission protein DRP1 (P-DRP1) were dramatically decreased in KO mice under physiological conditions. The protein levels of OPA1and P-DRP1 showed no differences in WT mice between the non-irradiated group and the irradiated group but had remarkably increased levels in the KO mice after irradiation. These results indicate that inhibition of autophagy reduces irradiation-induced subcortical white matter injury not by reducing inflammation, but by increasing mitochondrial fusion and inhibiting mitochondrial fission.

**Supplementary Information:**

The online version contains supplementary material available at 10.1007/s12035-021-02653-x.

## Background

Radiotherapy is an effective method for the treatment of malignant brain tumors in children; however, although the tumor cells are killed, such treatments can also damage normal brain tissue [1]. The developing brain in young children is much more sensitive to radiotherapy compared to the adult brain, and it is more prone to long-term complications such as cognitive impairment, endocrine hormone disorders, and secondary malignant tumors, which severely reduces the long-term quality of life in childhood survivors of malignant brain tumors [2–4]. Irradiation-induced brain injury consists of a series of pathophysiological changes that take place after neural cells and intracranial blood vessels are injured by radiotherapy. Studies have found that even when low-to-medium-dose irradiation is administered, more than 30% of children have delayed mental or behavioral development [5]. Therefore, the prevention of neural cell death induced by radiotherapy and its long-term complications is an urgent clinical issue.

Autophagy is essential for survival, differentiation, development, and homeostasis by removing damaged and harmful components in cells. However, the inappropriate activation of autophagy is involved in cell death in the immature brain and is associated with childhood neurological disorders [6]. A recent study showed that autophagy inhibitors could significantly alleviate the radiation-induced cerebral capillary damage and prolong the survival time in zebrafish larvae [7]. Another study found that radiation exposure led to the activation of autophagy in rat hippocampal neurons, and excessive activation of autophagy might damage synaptic plasticity by mediating synaptic vesicle degradation [8]. These studies provide evidence that excessive autophagy leads to capillary or synapse damage after irradiation, and autophagy inhibition might be a therapeutic target for irradiation-induced white matter injury. In our previous study, we showed that selective inhibition of autophagy in neural cells reduces irradiation-induced oligodendrocyte progenitor cell (OPC) loss [9]. However, how deficiency in autophagy prevents white matter injury after irradiation is still not clear, and further research is needed.

The aim of this study was to investigate how the genetic inhibition of autophagy in the juvenile mouse brain impacts irradiation-induced subcortical white matter injury. We found that selective neural autophagy inhibition reduced irradiation-induced subcortical white matter injury not by reducing inflammation, but by increasing mitochondrial fusion and inhibiting mitochondrial fission.

## Materials

### Animals and Ethical Permission

Floxed *Atg7* mice were characterized as previously described [6, 9] and were crossed with a nestin-Cre-driven line to produce *Atg7*^*flox/flox*; Nes−Cre^ knockout (*Atg7* KO) and *Atg7*^*flox/*+; Nes−Cre^ mice (WT). All of the mice were housed in a temperature-controlled and pathogen-free environment with a 12:12-h light–dark cycle. The genotyping of the pups was as described previously [6]. All experiments were approved by the animal research ethics committee (Gothenburg Committee of the Swedish Agricultural Agency) in accordance with national animal welfare legislation (2200–19).

### Irradiation Procedure

Postnatal day 10 (P10) *Atg7* KO and WT littermate pups of both sexes were anesthetized with a 50 mg/kg intraperitoneal injection of tribromoethanol (Avertin, Sigma-Aldrich, Stockholm, Sweden) and placed in a prone position (head to gantry) on a Styrofoam bed. The irradiation of the animals was performed using a linear accelerator (Varian Clinac 600CD; Radiation Oncology System LLC, San Diego, CA, USA) with 4 MV nominal photon energy and a dose rate of 2.3 Gy/min. The whole brain was irradiated with a single dose of 6 Gy to each mouse. The source-to-skin distance was approximately 99.5 cm. The head was covered with a 1-cm tissue-equivalent bolus material to obtain an even irradiation dose throughout the underlying tissue. After irradiation, the pups were returned to their dams and sacrificed at 5 days after irradiation. The sham-irradiated controls were anesthetized but not irradiated. We detected p62, which is used as a reporter of autophagy activity, by western blot in both irradiated and non-irradiated mouse brains at 5 days after irradiation. The results showed the p62 level was reduced by 41% at 5 days after irradiation; however, Atg7 increased by 36% at 5 days after irradiation, indicating that autophagy activity was elevated at this time point (Fig. S1).

### Immunohistochemistry Staining

The mouse pups were deeply anesthetized with 50 mg/ml phenobarbital and perfused intracardially with phosphate buffered saline (PBS) and 5% buffered formaldehyde (Histofix; Histolab, Gothenburg, Sweden) at 5 days after irradiation, and their brains were removed and fixed in 5% buffered formaldehyde at 4 °C for 24 h. After dehydration with graded ethanol and xylene, the brains were paraffin-embedded and cut into 5-μm sagittal sections. Every 50th section from one hemisphere was deparaffinized in xylene and rehydrated in graded ethanol concentrations and stained for myelin basic protein (MBP), platelet derived growth factor receptor α (PDGFRα), ionized calcium-binding adaptor molecule 1 (Iba-1), and glial fibrillary acidic protein (GFAP). Antigen retrieval was performed by heating the sections in 10 mM boiling sodium citrate buffer (pH 6.0) for 10 min. Nonspecific binding was blocked for 30 min with 4% donkey or goat serum in PBS for 30 min. The primary antibodies were mouse anti-MBP (1:1,000 dilution, BioLegend, SMI 94, 836,504), rabbit anti-PDGFRα (1:400 dilution, Cell Signaling, 3164), rabbit anti-Iba-1 (1:200 dilution, Wako Pure Chemical Industries, Ltd, 019–19,741), and mouse anti-GFAP (1:250 dilution, Cell Signaling, 3670). After incubating the sections with the primary antibodies overnight at 4 °C, the appropriate biotinylated secondary antibodies (1:200 dilution; all from Vector Laboratories, Burlingame, CA, USA) were added and incubated for 60 min at room temperature. After blocking endogenous peroxidase activity with 3% H_2_O_2_ for 10 min, the sections were visualized with Vectastain ABC Elite (Vector Laboratories) and 0.5 mg/mL 3,3’-diaminobenzidine enhanced with ammonium nickel sulfate, beta-D glucose, ammonium chloride, and beta-glucose oxidase. After dehydrating with graded ethanol and xylene, the sections were mounted using Vector mounting medium.

### Cell Quantification in Mice

Iba-1, PDGFRα, and GFAP-positive cells were counted in the subcortical white matter under a stereo microscope (MicroBrightField, Magdeburg, Germany). The counting area in the subcortical white matter was traced, and the number of cells was expressed as cells/mm^2^ [9, 10]. The counting was performed by a person who did not have prior knowledge of the groups. Three sections were counted with an interval of 250 μm between sections.

### White Matter Injury Evaluation and Volume Measurement

Four sections of each sample were used to measure the MBP-positive area using Micro Image (Olympus, Japan). The subcortical MBP-positive white matter volume (mm^3^) was calculated as previously described [11] using the following formula: V = SA × p × T, where V is the total volume, SA is the sum of the areas measured, p is the inverse of the section sampling fraction, and T is the section thickness. To analyze cortical myelination as an indication of myelinated axons, the length of myelinated fibers within the cortex was measured between the end of the myelinated axons and the cortical plate at fixed levels using the ImageJ software [11]. The immunodensity of MBP-positive staining in the myelinated axons was determined using the Image J software and manually setting the threshold to include the MBP-stained area followed by measuring the proportion of the field that was positive for MBP staining in the subcortical white matter. The MBP immunodensity was determined by measuring the integrated density and normalizing it to the WT non-irradiated group [9, 11].

### RNA Isolation, cDNA Synthesis, and RNA Sequencing

Samples of subcortical white matter from WT and KO mice after irradiation at 5 days and from the non-irradiated group were prepared for RNA isolation and RNA sequencing. Total RNA was isolated using the RNeasy mini kit (Qiagen, 74,104) according to the manufacturer’s instructions. The concentration and purity of all RNA samples were determined using a Nanodrop spectrophotometer (Nanodrop Technologies, Wilmington, DE, USA). The integrity of RNA was measured using the Experion RNA StdSens analysis kit (Bio-Rad, 7,007,103) on an Automated Electrophoresis Station (Bio-Rad, Hercules, CA, USA). One microgram of total RNA was reverse transcribed using the QuantiTect Reverse Transcription kit (Qiagen, 205,311).

RNA was sent for sequencing to Novogene, UK. In brief, quality control of sample purity and integrity was performed using a Nanodrop and Agilent 2100. And the libraries were sequenced on an Illumina NovaSeq platform to generate 150 bp paired-end reads, according to the manufacturer’s instructions. Mapping to the reference genome was performed using HISAT2 software and quantification was performed using FeatureCounts software [12]. The DESeq method was used to screen for differences between the two groups according to the criterion of adjusted *p* < 0.05. Principal component analysis (PCA) was performed to identify the variability and repeatability of the samples, and a volcano plot was used to visualize the overall distribution of differentially expressed genes using *ggplot2* in the R package (version 3.3.3). Gene ontology (GO) term classification and Gene Set Enrichment Analysis (GSEA) analysis were performed using the cluster Profiler R package [13] and MITOCARTA 3.0 (https://www.broadinstitute.org/mitocarta/mitocarta30-inventory-mammalian-mitochondrial-proteins-and-pathways) as the reference genome for mitochondria-related genes.

### Quantitative Real-Time PCR

Quantitative real-time PCR (qRT-PCR) was performed using a LightCycler 480 instrument (Roche Diagnostics, Germany) and the SYBR Green technique according to the manufacturer’s instructions. The primers used in the qRT-PCR reactions were designed by Beacon Designer software (free trial, PREMIER Biosoft) and included the oligodendrocyte and myelin-related genes *Olig2* (sense: 5’-CGG TGG CTT CAA GTC ATC-3’, antisense: 5’-GTC ATC TGC TTC TTG TCT TTC T-3’), *Cldn11* (sense: 5’-TGG CAT CAT CGT CAC AAC-3’, antisense: 5’-AGC CCA GTT CGT CCA TTT-3’), *CNP* (sense: 5’-TCT ACT TTG GCT GGT TCC T-3’, antisense: 5’-CTT CTC CTT GGG TTC ATC TC-3’), and *MBP* (sense: 5’-CCT CAC AGC GAT CCA AGT-3’, antisense: 5’-CAA GGA TGC CCG TGT CTC-3’); the microglia-related gene *CX3CR1* (sense: 5’-GTC TGG TGG GAA ATC TGT TG-3’; antisense: 5’-GGC TGA TGA GGT AGT GAG T-3’); and the astrocyte-related genes *GFAP* (sense: 5’-GAG GTG GAG AGG GAC AAC-3’, antisense: 5’-TCT ATA CGC AGC CAG GTT-3’) and *Vimentin* (sense: 5’-TTC AAG ACT CGG TGG ACT-3’, antisense: 5’-GCA GTT CTA CCT TCT CGT T-3’). The mitochondrial fusion and fission-related genes included *Opa1* (sense: 5’-CCT GTG AAG TCT GCC AAT-3’, antisense: 5’-TTA GAG AAG AGA ACT GCT GAA AT-3’), *Drp1* (sense: 5’-TGC TCA GTA TCA GTC TCT TC-3’, antisense: 5’-GGT TCC TTC AAT CGT GTT AC-3’), and *Fis1* (sense: 5’-ATG AAG AAA GAT GGA CTG GTA G-3’, antisense: 5’-GGA TTT GGA CTT GGA GAC A-3’). The reference genes were *Sdha* (sense: 5’-TTG CCT TGC CAG GAC TTA-3’, antisense: 5’-CAC CTT GAC TGT TGA GAA T-3’) and *B2m* (sense: 5’-CCG AAC ATA CTG AAC TGC TA-3’, antisense: 5’-AGG ACA TAT CTG ACA TCT CTA CTT-3’). The relative mRNA expression levels were calculated by the method of geometric averaging of multiple internal control genes.

### Multiplex Cytokine/Chemokine Assay

Cytokines and chemokines were measured in subcortical white matter homogenate supernatant fractions at 5 days after irradiation. Protein concentrations were measured with the BCA protein assay (Sigma, A2058) using samples prepared according to the manufacturer’s protocol. Levels of interleukin (IL)-1β, IL-2, IL-4, IL-6, IL-10, and keratinocyte-derived chemokine (KC) were measured simultaneously using the Luminex Multiplex Cytokine Assay (Merck Chemicals and Life Science AB). The results were normalized to the total amount of protein in the sample.

#### **Immunoblotting**

Protein concentration was determined using the bicinchoninic acid method. Subcortical white matter homogenate samples (65 µl) were mixed with 25 µl NuPAGE LDS 4 × sample buffer (Thermo Fisher Scientific, NP0007) and 10 µl reducing agent (ThermoFisher Scientific, NP0004) and heated at 70 °C for 10 min. Samples were run on 4–12% NuPAGE Bis–Tris gels (Invitrogen) and transferred to reinforced nitrocellulose membranes (Bio-Rad). After blocking with 5% fat-free milk in TBST buffer (20 mM Tris, 150 mM NaCl, and 0.1% Tween 20, pH 7.6) for 60 min at room temperature, the membranes were incubated with the following primary antibodies: mouse anti-OPA1 (1:1000 dilution, mouse monoclonal antibody, 612,606, BD Bioscience), mouse anti-DRP1 (1:500 dilution, mouse monoclonal antibody, sc-271583, Santa Cruz Biotechnology), rabbit anti-phospho-DRP1 (Ser637) (1:1000 dilution, rabbit polyclonal antibody, 4867S, Cell Signaling), rabbit anti-FIS1 (FL-152) (1:500 dilution, rabbit polyclonal antibody, sc-98900, Santa Cruz Biotechnology), mouse anti-PINK1 (38CT20.8.5) (1:500 dilution, mouse monoclonal antibody, sc-517353, Santa Cruz Biotechnology), rabbit anti-Atg7 (1:500 dilution, rabbit polyclonal antibody, ab223380, Abcam), rabbit anti-Sequestosome 1 (SQSTM1/p62) (1:1000 dilution, rabbit monoclonal antibody, Cell Signaling Technology, 23,214), mouse anti-Total OXPHOS Rodent western blot antibody (1:250 dilution, Abcam, ab110413), mouse anti-VDAC1 (1:500 dilution, Santa Cruz, sc-390996), or rabbit anti-Actin (1:200 dilution, rabbit polyclonal antibody, A2066, Sigma) overnight. After washing, the membranes were incubated with peroxidase-labeled goat anti-rabbit IgG antibody (1:2000 dilution, Vector, PI-1000) or peroxidase-labeled horse anti-mouse IgG antibody (1:4000 dilution, Vector, PI-2000). Immunoreactive species were visualized using the SuperSignal West Pico PLUS Chemiluminescent Substrate (ThermoFisher Scientific, 34,580) and an LAS 3000 cooled CCD camera (Fujifilm, Japan).

### Statistical Analysis

The Statistical Package for the Social Sciences 17.0 (SPSS, IBM, NY, USA) was used for all analyses. Comparisons between groups were performed by Student’s *t*-test, and data with unequal variance were compared with the Mann–Whitney *U*-test. Two-way ANOVA followed by a Bonferroni post hoc test was used for multiple comparison correction of data from more than two groups. Results are presented as means ± SEM, and *p* < 0.05 was considered statistically significant.

## Results

### Disruption of Subcortical White Matter Development in Mice After Irradiation

**Fig. 1:**
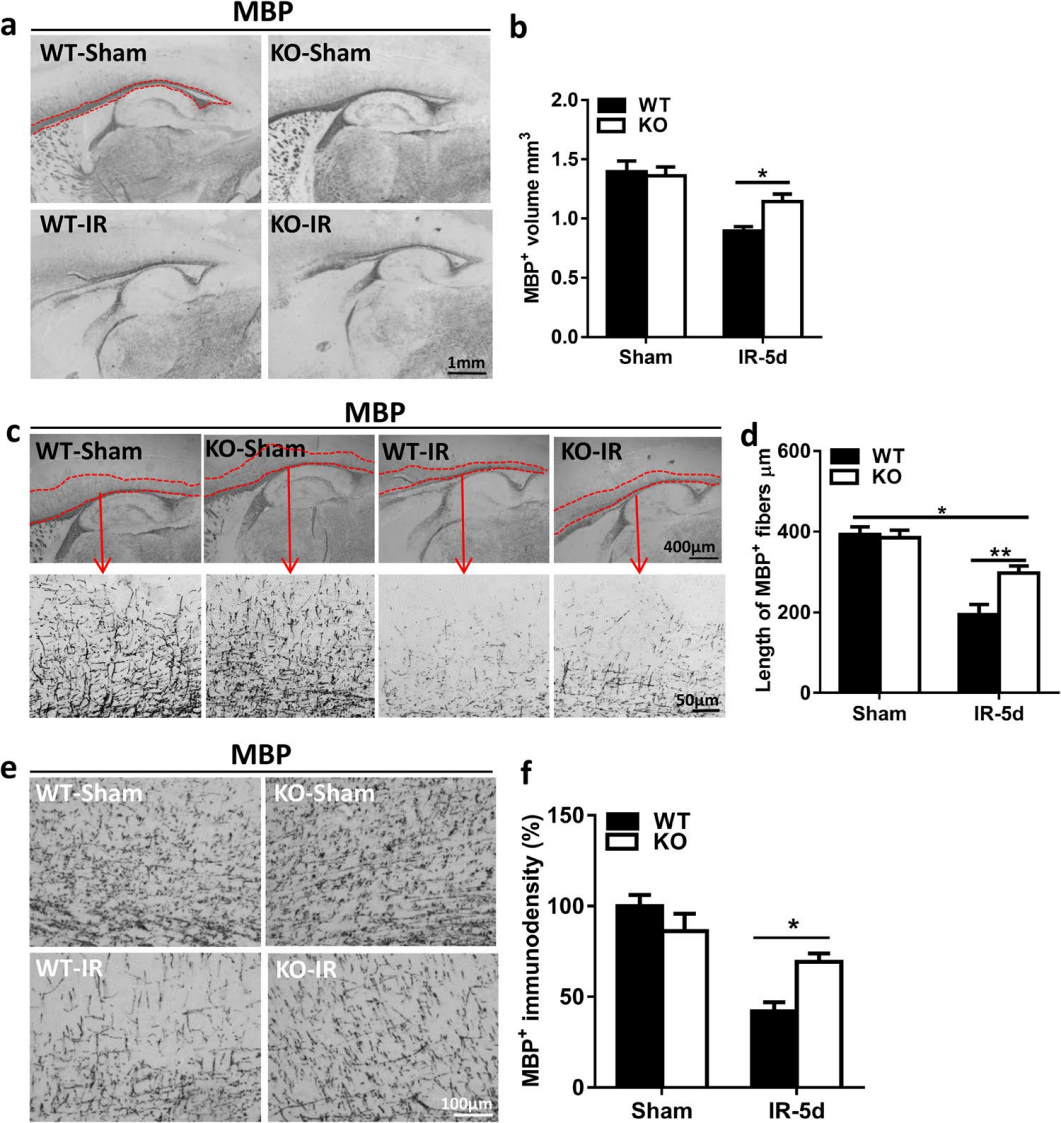
Cerebral irradiation interrupts subcortical white matter development in young mice **a** Representative sagittal hemisphere MBP staining in WT-Sham and *Atg7* KO-Sham and irradiated mouse pups. The red line represents the subcortical white matter. **b** The subcortical white matter volume assessed as the volume of MBP-positive staining. *Atg7* KO protected against the irradiation-induced reduction in subcortical white matter. **c** Representative MBP staining in subcortical white matter and myelinated fibers. The red line represents myelinated fibers within the cortex. **d** The mean lengths of MBP-positive myelinated fibers between the end of myelinated axons and the cortical plate at fixed levels. **e** Representative MBP immunostaining in myelinated fibers within the cortex. **f** The MBP-positive immunodensity in myelinated fibers presented as the percentage of controls. *n* = 7/group for the immunostaining. *p < 0.05, **p < 0.01.

Myelination was visualized in the hemispheres of the brain as indicated by MBP staining (Fig. 1a). Subcortical white matter volume was assessed as the MBP-positive volume, and the volume was significantly reduced in WT mice (0.90 ± 0.04 mm^3^) after irradiation compared to *Atg7* KO mice (1.14 ± 0.06 mm^3^) (*p* = 0.037) (Fig. 1b). The irradiated mice also showed significantly shorter myelinated fibers within the cortex compared to non-irradiated mice (Fig. 1c), but *Atg7* deficiency protected against the shortening of myelinated fibers after irradiation (the mean fiber length was 296.9 μm in KO mice vs. 194.7 μm in WT mice, *p* = 0.003, Fig. 1d). Further analysis of the MBP-positive immunodensity in the myelinated fibers within the cortex showed that *Atg7* deficiency caused less obvious myelin disruption compared to the WT littermates after irradiation (Fig. 1e, f) (*p* = 0.016).

### Atg7 Deficiency Reduces Irradiation-Induced OPC Loss

**Fig. 2:**
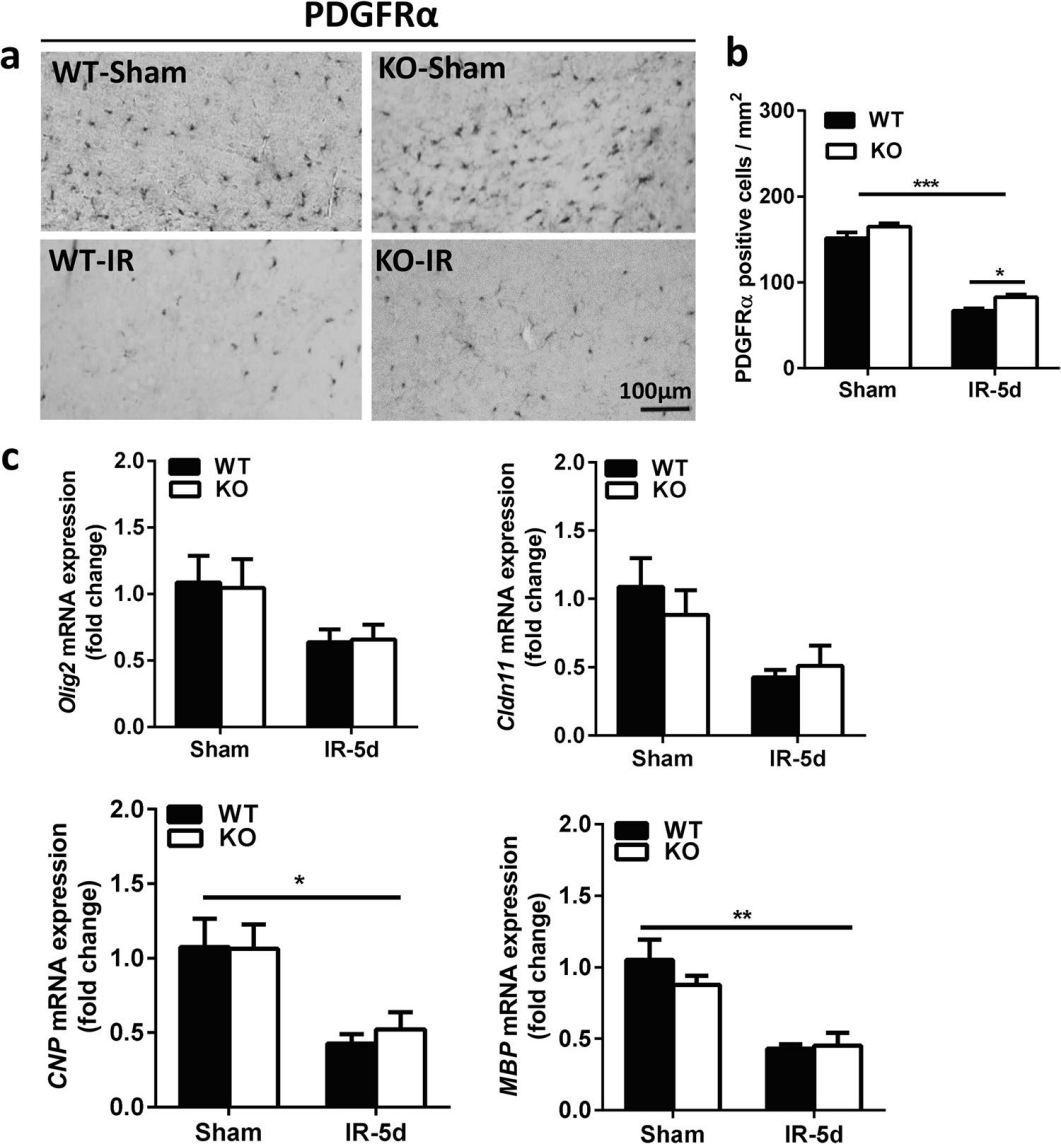
*Atg7* KO reduces irradiation-induced OPC loss in the subcortical white matter **a** Representative images of OPCs in the subcortical white matter after irradiation that were immunostained for PDGFRα in WT-Sham and *Atg7* KO-Sham and irradiated mouse pups. **b** Quantitative analysis of the PDGFRα-labeled cells in the subcortical white matter. **c** Bar graphs showing the mRNA expression of *Olig2*, *Cldn11*, *CNP*, and *MBP* in the cortical tissue, including the subcortical white matter, at 5 days after irradiation. *n* = 7/group for the immunostaining, and *n* = 5/group for qRT-PCR. *p < 0.05, **p < 0.01, ***p < 0.001.

As a marker of OPCs, we used PDGFRα staining to determine the extent of irradiation-induced white matter injury [9]. PDGFRα-positive cells were located mainly in the subcortical white matter (Fig. 2a), and the numbers of PDGFRα-positive cells were much lower in both groups of mice after irradiation (*p* < 0.001). However, there were more PDGFRα-positive cells in the *Atg7* KO mice compared with WT mice at 5 days after irradiation (*p* = 0.033) (Fig. 2b). We also measured the mRNA expression of oligodendrocyte-related and myelin-related genes (Fig. 2c). *Olig2*, *Cldn11*, *CNP*, and *MBP* mRNA expression was slightly higher in the *Atg7* KO mice compared to WT mice after irradiation, but there was no significant difference between *Atg7* KO and WT pups.

### Microglia Activation and Astrocyte Reactivity After Irradiation

**Fig. 3:**
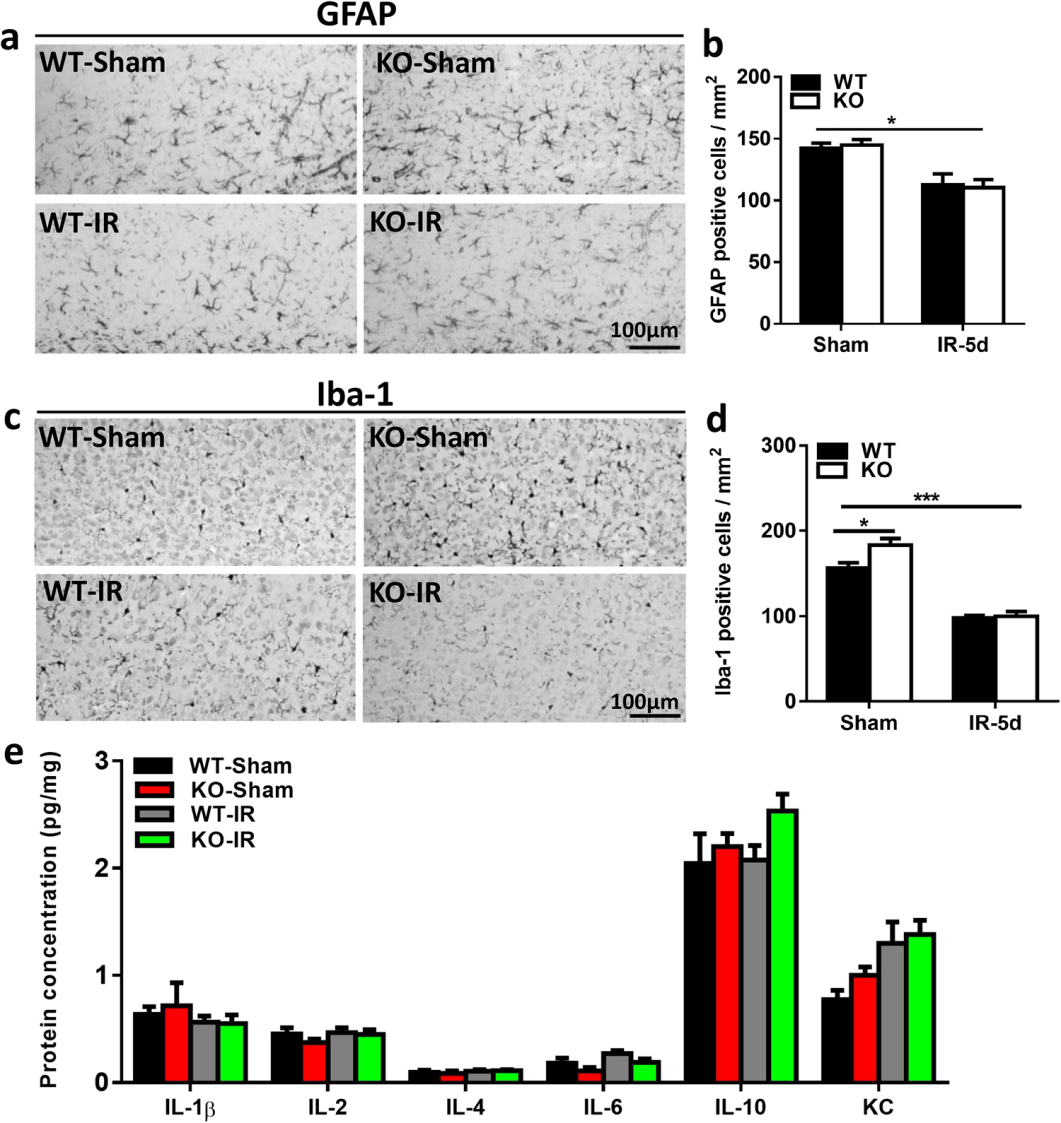
Astrocyte and microglia changes in the subcortical white matter after irradiation **a** Representative images of GFAP-labeled cells in the subcortical white matter. **b** Quantification of GFAP-labeled cells in the subcortical white matter. **c** Representative Iba-1 immunostaining in the subcortical white matter. **d** Quantification of Iba-1–labeled cells in the subcortical white matter. **e** The protein levels of IL-1β, IL-2, IL-4, IL-6, IL-10, and KC in the cortical tissue at 5 days after irradiation as detected by Luminex assay in the *Atg7* KO and WT pups. *n* = 7/group for the immunostaining and Luminex assay. *p < 0.05, ***p < 0.001.

Microglia activation and astrocyte reactivity are related to chronic inflammation in the brain, and GFAP-labeled cells, as an indicator of astrocytes, were observed in the subcortical white matter (Fig. 3a). The number of astrocytes was significantly reduced after irradiation, but there was no significant difference between KO and WT pups (Fig. 3b). Similarly, Iba-1 labeling, as a marker of microglia, was also significantly reduced at 5 days after irradiation but showed no significant difference between KO and WT pups (Fig. 3c, d). We measured the expression of the microglia-related gene *CX3CR1* (Fig. S2a) and the astrocyte-related genes *GFAP* and *Vimentin* (Fig. S2b-2c), and we observed no significant changes between KO and WT mice. We also measured the protein levels of IL-1β, IL-2, IL-4, IL-6, IL-10, and KC in the subcortical white matter and found no difference at 5 days after irradiation between the *Atg7* KO and WT groups (Fig. 3e).

### Irradiation Induces Transcriptome Alterations in *Atg7* KO and WT Mice

**Fig. 4:**
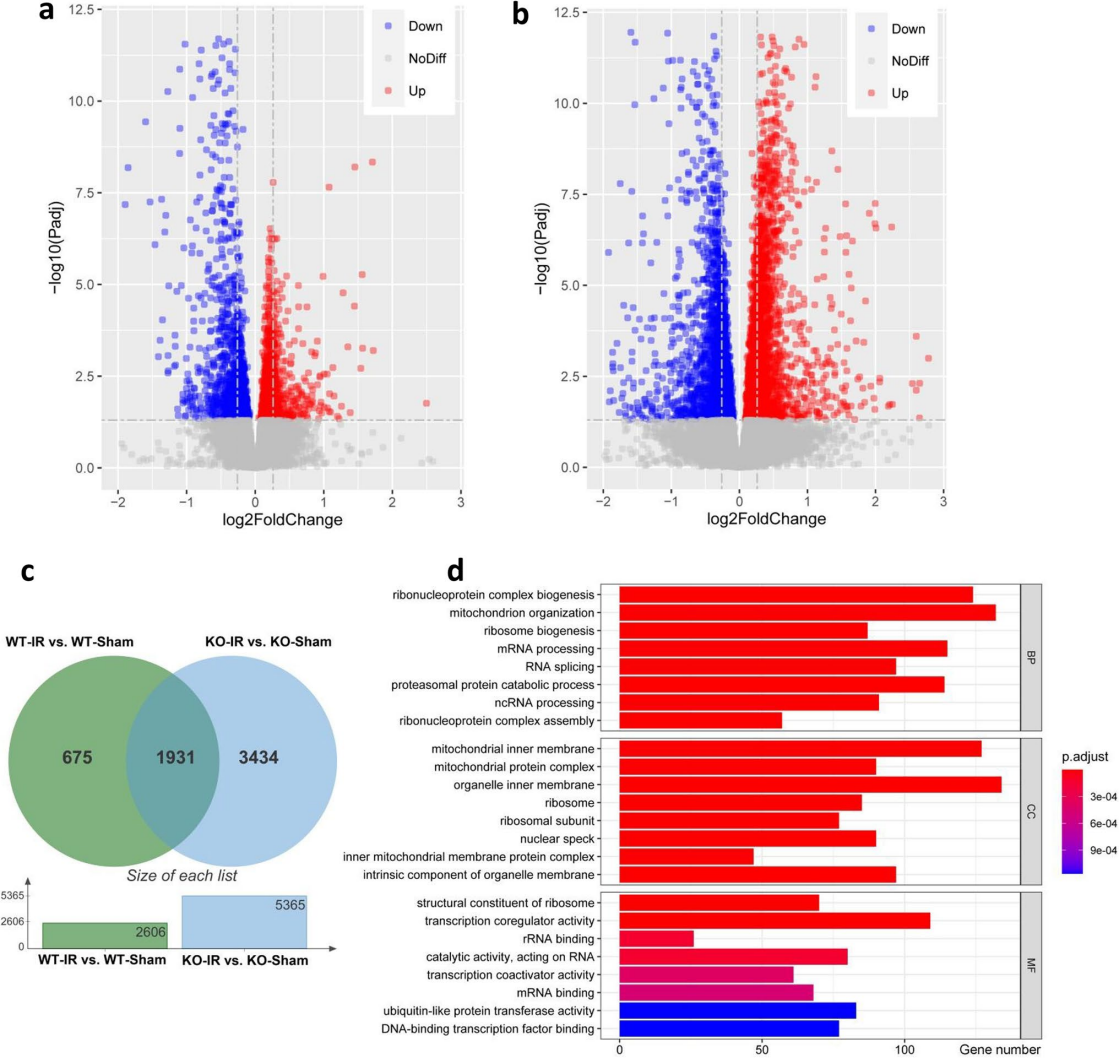
Irradiation induces transcriptome alterations in WT and *Atg7* KO mice **a** Volcano plot showing DEGs between WT irradiated mice and WT non-irradiated mice in the subcortical white matter. **b** Volcano plot showing DEGs between KO irradiated mice and KO non-irradiated mice in the subcortical white matter. **c** Venn diagram showing the intersection of DEGs between *Atg7* KO and WT mice after irradiation. **d** The graph shows the top eight classified GO terms in three ontologies. GO classification was performed based on the 1931 DEGs. The *x*-axis represents the number of DEGs, and the *y*-axis represents the GO terms. MF, molecular biological function; CC, cellular component; BP, biological process. *n* = 6/group for RNA sequencing.

To further investigate whether *Atg7* KO has an impact on the mRNA transcriptome under physiological conditions or after irradiation, the transcriptomic profiles of six *Atg7* KO and WT mouse brain tissue samples from the subcortical white matter were determined by RNA sequencing. Even when using the relaxed criterion of *p* < 0.05, the data showed that 2606 of the total of 17,747 genes were differentially expressed in WT irradiated mice compared to WT non-irradiated mice. Among these 2606 genes, 1274 were upregulated and 1332 were downregulated (Fig. 4a). For *Atg7* KO mice, the data analysis showed that 5365 of the total of 20,753 genes were differentially expressed in the irradiated group compared to the non-irradiated group. Among these 5365 genes, 2916 were upregulated and 2449 were downregulated (Fig. 4b). To compare the irradiation-induced differentially expressed genes (DEGs) between WT and *Atg7* KO mice, Venn plot analysis was performed, and 1931 DEGs were found in both WT and *Atg7* KO mice after irradiation (Fig. 4c). GO term classification was performed on the 1931 DEGs in three ontologies (molecular biological function, cellular component, and biological process) (Fig. 4d). Among the top eight classified GO terms according to the adjusted *p*-value, we found that most of the terms were non-specific, but some of them were related to mitochondria.

### Irradiation Induces Changes in the Expression of Mitochondria-Related Genes

**Fig. 5:**
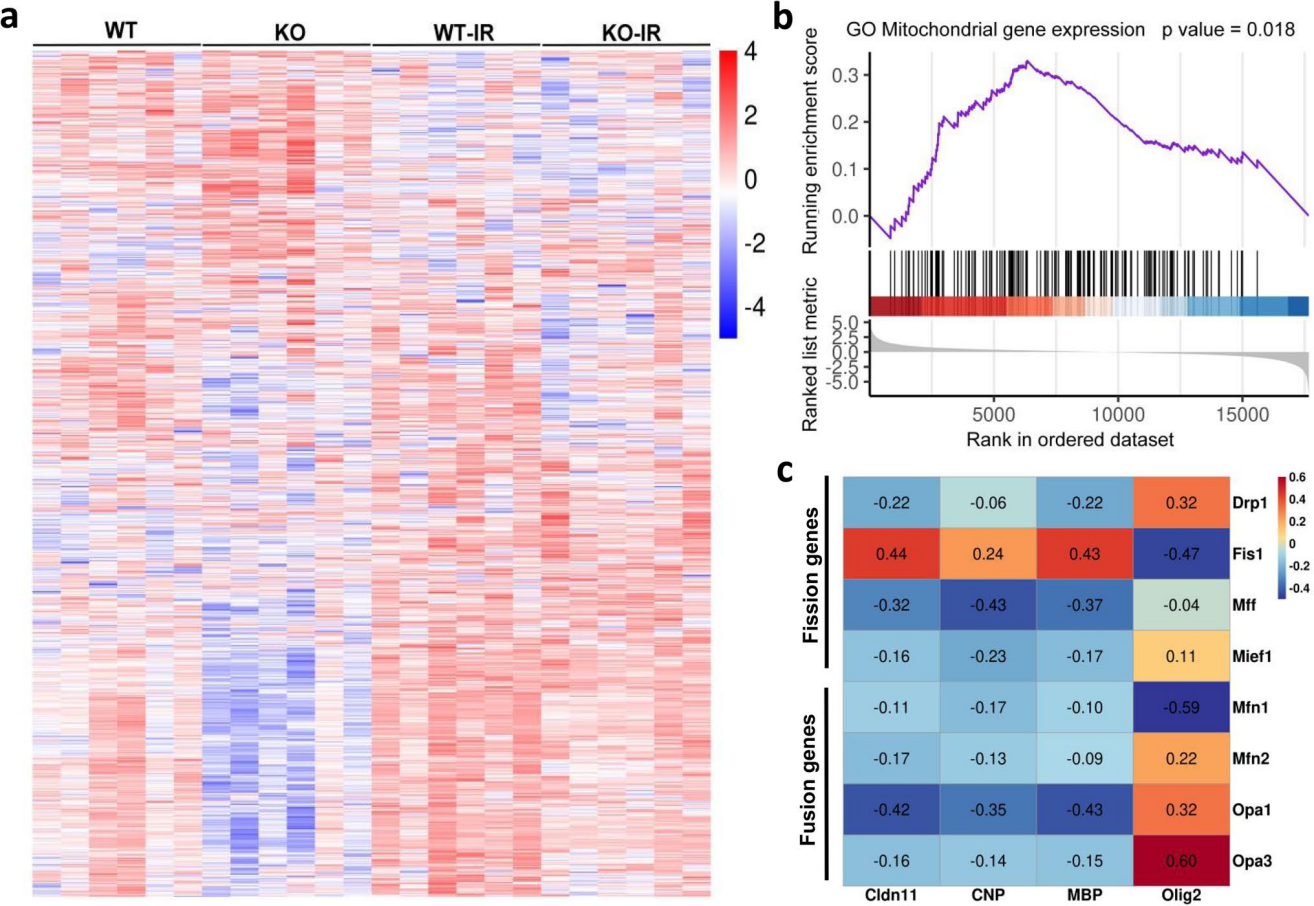
Irradiation induces mitochondria-related gene alterations **a** Heatmap showing the overall distribution of 1109 mitochondria-related genes. **b** GSEA analysis showed DEGs between WT-irradiated mice and KO-irradiated mice in the GO Mitochondrial gene expression pathway. The top part of each plot shows the enrichment score that represents running-sum statistics calculated by “walking down” the ranked list of genes. The middle part shows the position of a member of a gene set in the ranked list of genes. The bottom part depicts the ranking metric that measures a gene’s correlation with a biological function. **c** Correlation heatmap for mitochondria fusion and fission-related genes and oligodendrocyte and myelin-related genes between WT-irradiated mice and KO-irradiated mice. Some genes were negatively related and others were positively related as represented in different colors, with red representing positive correlation and blue representing negative correlation. The number in the correlation heatmap is the correlation coefficient.

Previous studies showed that mitochondria-related genes are more likely to specifically be involved in irradiation-induced brain injury [14, 15], and 1109 mitochondria-related genes in both KO and WT mice under physiological conditions or after irradiation were identified using MITOCARTA 3.0 (Fig. 5a). For further comparing the mitochondria-related gene expression between the KO and WT groups after irradiation (WT-irradiated mice vs KO-irradiated mice), GSEA analysis showed significant differences (*p* = 0.018) in the DEGs in the GO Mitochondria gene expression pathway (Fig. 5b). Correlation analysis was performed for mitochondrial fusion (*Mfn1*, *Mfn2*, *Opa1*, and *Opa3*) and fission (*Drp1*, *Fis1*, *Mff*, and *Mief1*)-related genes and for oligodendrocyte and myelin-related genes (*Cldn11*, *CNP*, *MBP*, *Olig2*) (Fig. 5c). The correlation heatmap showed that the *Cldn11*, *CNP*, and *MBP* genes had more negative correlations with mitochondrial fusion and fission genes, while the *Olig2* gene had more positive correlations with mitochondrial fusion and fission genes.

### Effect of Atg7 Deficiency on Mitochondrial Fission and Fusion After Irradiation

**Fig. 6:**
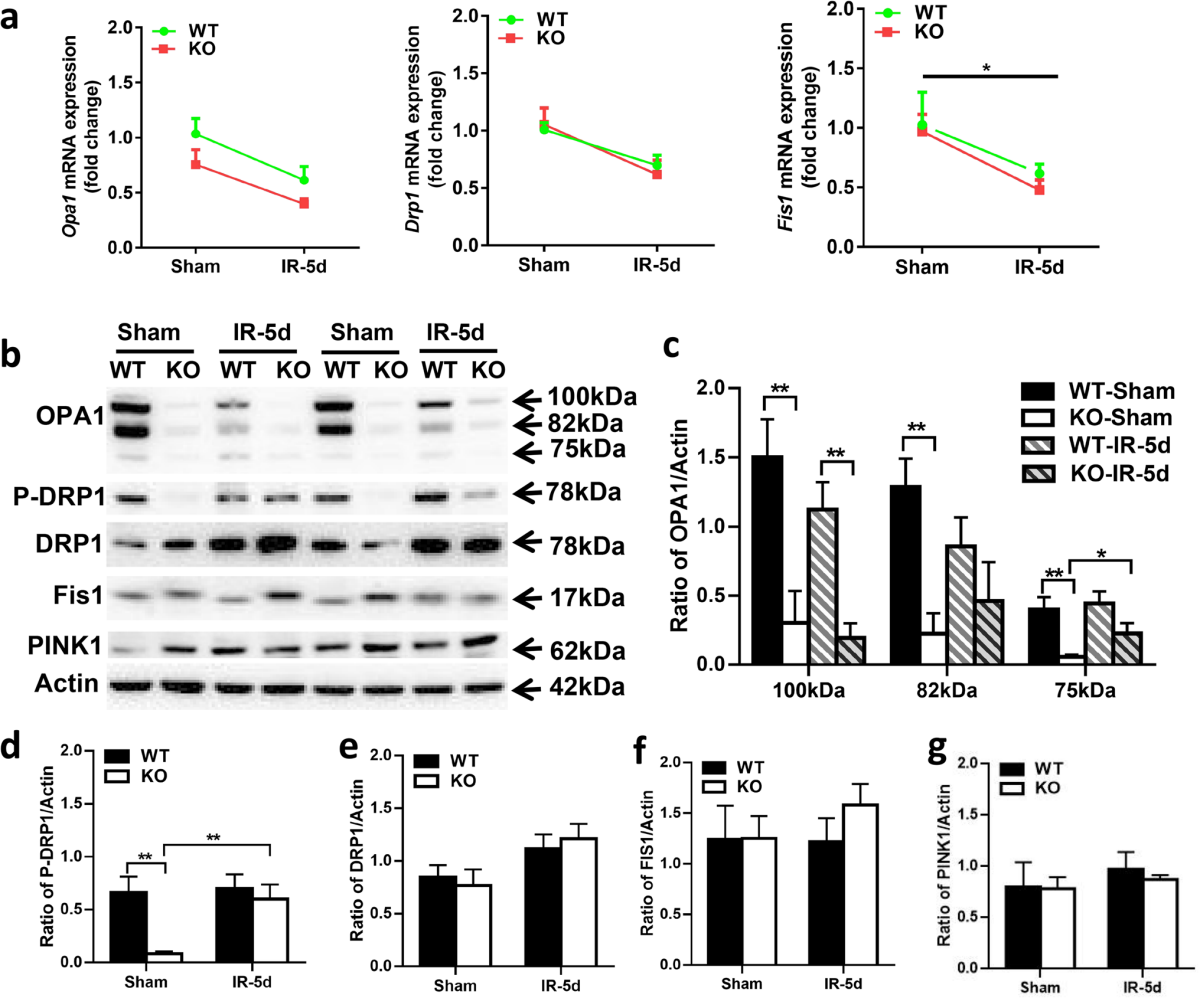
Effect of *Atg7* deficiency on mitochondrial fission and fusion in the subcortical white matter after irradiation **a** Bar graphs showing mRNA expression of *Opa1*, *Drp1*, and *Fis1* at 5 days after irradiation. **b** Representative immunoblots of mitochondrial fusion protein (OPA1), fission proteins (P-DRP1/DRP1 and Fis1), and mitophagy-related protein PINK1 in WT and KO mice under physiological conditions and after irradiation. **c–g** Quantification of OPA1, P-DRP1, DRP1, FIS1, and PINK1. *n* = 5/group for qRT-PCR, *n* = 6/group for immunoblotting. *p < 0.05, **p < 0.01.

Based on the degree of correlation between mitochondrial fusion-fission genes and oligodendrocyte-myelin genes, the expression levels of *Opa1*, *Drp1*, and *Fis1* were confirmed by qRT-PCR. The expression of *Opa1*, *Drp1*, and *Fis1* was reduced after irradiation, but there were no significant differences between KO and WT either under physiological conditions or after irradiation (Fig. 6a). Western blot was performed to confirm the protein levels of OPA1, DRP1, and FIS1 (Fig. 6b), and the quantitative protein expression showed that the OPA1 protein level was significantly reduced in KO mice compared with WT mice in the non-irradiated groups (*p* < 0.01), while in the irradiated groups, the OPA1 protein expression was higher in WT mice. However, WT mice after irradiation expressed less OPA1 protein at 100 kDa and 82 kDa, and a similar level at 75 kDa, while KO mice after irradiation expressed less OPA1 protein at 100 kDa, but higher protein levels at 82 kDa and significantly increased levels at 75 kDa (*p* = 0.047) (Fig. 6c).

Phosphorylated DRP1 (P-DRP1) at Ser637 by protein kinase A has been shown to cause a significant decrease in GTPase activity and to inhibit mitochondrial fission [16, 17]. Compared with WT mice, the abundance of P-DRP1 in KO mice was significantly decreased under physiological conditions (*p* < 0.01) but only slightly reduced after irradiation. Interestingly, the level of P-DRP1 showed no difference in WT mice between the non-irradiated group and the irradiated group but was sharply increased in KO mice after irradiation (*p* = 0.004) (Fig. 6d). We found no difference in DRP1, FIS1, or PINK1 protein levels (Fig. 6e–g).

Mitophagy plays critical roles in mitochondrial biogenesis, fusion, fission, and degradation and in removing damaged mitochondrial DNA (mtDNA) in order to maintain respiratory function. The effect of irradiation on individual mitochondrial respiratory chain complex (C-I, C-II, C-III, C-IV, C-V) subunit expression and mitochondria membrane protein VDAC1 is shown in Fig. S3a-3c, as well as the mtDNA copy number changes in *Atg7* KO and wildtype groups at 5 days after irradiation (Fig. S3d).

## Discussion

Autophagy is a catabolic process that breaks down and recycles unnecessary or damaged cellular components, and it plays essential roles in development and in maintaining homeostasis in organisms [18]. As a classic autophagy-related gene, *Atg7* is crucial for the assembly and function of ubiquitin-like conjugates in the expansion of autophagosomal membranes [19]. Recent studies have shown that Atg7 is involved in the regulation of a variety of brain injury animal models [6, 20, 21], suggesting that Atg7 may be a target for therapeutic interventions. The role of autophagy is quite different between the adult and juvenile brain. For example, autophagy might have a neuroprotective effect in the adult brain under conditions of acute neurotoxicity, traumatic damage and stroke, and neurodegenerative or aging-related diseases [22–24]. Autophagy-mediated neurotoxicity was reported in the neonatal and juvenile brain after hypoxic-ischemic brain injury or irradiation-induced brain injury [6, 9]. The impact of autophagy on certain forms of brain damage varies depending on the type of stress and the developmental stage of the brain as well as other factors, and thus the roles and mechanisms of autophagy in the adult and juvenile brain under different pathophysiological models remain to be more precisely elucidated.

In our previous studies, we demonstrated that selective *Atg7* deletion prevents irradiation-induced caspase-3 activation, microglia activation, and inflammation and reduces irradiation-induced neural stem and progenitor cell death during the acute injury phase [10]. We also showed that selective inhibition of autophagy in neural cells reduces irradiation-induced subacute cerebellar white matter injury by decreasing OPC loss [9]. However, the mechanism through which autophagy deficiency prevents white matter injury after irradiation in the subacute injury phase is still unclear, and further research is needed. In this study, we showed that inhibition of autophagy reduces irradiation-induced subacute subcortical white matter injury not by reducing inflammation, but by increasing mitochondrial fusion and inhibiting mitochondrial fission.

Irradiation-induced brain injury is characterized by massive neural stem cell death followed by changes in cell metabolism, the cellular microenvironment, cell proliferation, and tissue shape as well as long-term cognitive impairments and growth reduction [25]. Studies of brain irradiation in animals have demonstrated the loss of myelin sheaths with apparent preservation of axons [26, 27], and this results in inhibited white matter development after irradiation as indicated by an up to 70% reduction in MBP staining [28]. The proliferation and differentiation of OPCs is critical for the development of oligodendrocytes, and these cells undergo myelination throughout life, thus making them susceptible to irradiation insult [29]. OPC loss occurs in the acute phase after irradiation, and the numbers of OPCs are reduced significantly by 2–4 weeks after irradiation, but these numbers recover by 6 weeks after irradiation [30]. Our previous study showed that *Atg7* deficiency reduced the severity of cerebellar white matter injury at 5 days after irradiation, and in the present study, we found that deficiency in neural autophagy also protects against radiation-induced subcortical white matter injury by preventing OPC loss. In order to clarify the relationship between inhibition of autophagy and neuroinflammation, we analyzed microglia and astrocytes as well as inflammatory cytokines.

Microglia are the resident phagocytes of the central nervous system, and they are involved in the maintenance of brain homeostasis and immune defense [31]. Astrocytes play important roles in maintaining the homeostasis of ions, transmitters, water, and blood flow that is critical for the proper functioning of neural circuits [32]. Microglia activation and astrocyte reactivity are related to inflammation in the brain after cerebral insults by causing the release of inflammatory chemokines and cytokines resulting in long-lasting chemical inflammatory brain injury [33, 34]. Studies have shown that pharmacological and starvation modulation of autophagy or knocking out ATG factors can enhance IL-1β secretion, which is a key pro-inflammatory cytokine processed by the inflammasome [35–37]. This facilitates unconventional secretion of the cytosolic cargo and involves autophagy, and is thus called secretory autophagy, because unlike proteins endowed with the leader peptides, these proteins cannot enter the conventional secretory pathway normally operating via the endoplasmic reticulum and the Golgi apparatus, and this process may also export more complex cytoplasmic cargo and help excrete particulate substrates [38]. In our previous study, we showed that proinflammatory cytokine IL-1β was reduced in the *Atg7* KO mice after irradiation at 6 h compared to the WT mice, but there was no difference between *Atg7* KO mice and WT mice under physiological conditions [10], which indicated that secretory autophagy might not be the main factor behind the reduced secretion of IL-1β in *Atg7* KO mice after irradiation. In addition, the concept of “secretory autophagy” is still unclear, and many questions remain to be answered before we can define secretory autophagy as a specific pathway [38]. Autophagy has been shown to regulate the severity of inflammation through several pathways [39]. Normally, autophagy reduces inflammation by clearing damaged mitochondria or inflammatory molecules and plays roles in attenuating excessive inflammatory activity by removing aggregated inflammasome components [40, 41]. Autophagy also inhibits inflammatory signal transduction pathways and influences the secretion of inflammatory cytokines and chemokines [42–44]. However, due to the heterogeneity of experimental factors (including the type, intensity, and duration of different stress stimuli and the sex and developmental stage of the animal, among others), autophagy has quite different effects on stress-induced inflammation [24]. Thus, it is important to investigate the relationship between autophagy and inflammation, which needs to be considered according to the specific experimental model and the type of stress and other factors. In the current mouse model, we found that microglia and astrocytes do not play a key role in irradiation-induced subcortical white-matter injury as indicated by cell markers and cytokines and chemokine levels, and this might be related to the time point when the blood–brain barrier (BBB) was disrupted after irradiation. In a juvenile mouse model study, the mice were given a single dose of 8 Gy irradiation, and the authors found no evidence that the BBB was damaged at 6 h, 24 h, or 7 days after irradiation [45]. In another study that reported on young mice receiving low to medium doses of irradiation, they showed that the permeability of the BBB increased significantly after 4 weeks of irradiation [46]. These previous findings provide evidence that the permeability of the BBB changes with time after irradiation, indicating that the BBB is relatively intact at 5 days after irradiation in this current model. Therefore, the cytokines secreted by peripheral inflammatory cells might not enter the CNS through the BBB and thus might not aggravate neuroinflammation.

Studies have suggested that irradiation-induced cognition dysfunction involves oxidative stress and inflammation, but the detailed mechanism remains unknown [25, 47]. Hao et al. found that autophagy could be over-activated in hippocampal neurons, resulting in reduced synaptic plasticity, and this was related to radiation-induced cognitive impairment [8]. Thus, autophagy might be a possible modulator for irradiation-induced cognitive dysfunction, although there is no direct evidence in the literature that inhibition of autophagy modulates such cognitive dysfunction. However, because irradiation induces autophagy and alters oxidative stress activity [48, 49], and because oxidative stress is involved in synaptic dysfunction and cognitive impairment [50, 51], we assume that the regulation of autophagy will modulate irradiation-induced cognitive dysfunction. Of course, further research is needed to determine if inhibiting autophagy has an impact on cognition at longer time points after irradiation.

To further investigate the mechanism through which *Atg7* deficiency prevents subcortical white matter injury, we performed a transcriptome analysis. We originally compared the DEGs between WT-irradiated mice and KO-irradiated mice, but there were only a few DEGs between WT-IR and KO-IR mice (258 DEGs), and the results showed there were a great amount of DEGs between WT-IR and WT-Sham mice (2606 DEGs) or KO-IR and KO-Sham mice (5365 DEGs) (Fig. S4a-4c). The functions of the DEGs between Atg7 KO-IR and WT-IR mice were predicted using GO and KEGG analyses (Fig. S4d), which showed that most of the terms were non-specific pathways. To our understanding, irradiation-induced brain injury might greatly affect the difference in DEGs and exceed the difference between WT-Sham and KO-Sham themselves, leading to some useful transcriptomic information being lost. Thus, we chose to show the DEGs between non-irradiated mice and irradiated mice and identified the co-expressed DEGs and involved pathways. Previous studies showed that irradiation might lead to metabolic alterations in mitochondria, and thus mitochondria-related genes are more likely to be specifically involved in irradiation-induced brain injury [14, 15]. In addition, mitochondria are dynamic organelles that produce energy and molecular precursors that are essential for myelin synthesis, and thus the regulation of mitochondrial dynamics is likely to be important in the physiology and pathology of myelinated axons [52]. We therefore analyzed all of the mitochondria-related genes in the both KO and WT mice under physiological conditions and after irradiation, and we found that DEGs in the GO Mitochondria gene expression pathway showed significant differences between the KO and WT groups after irradiation, and subsequent correlation analysis showed that oligodendrocyte and myelin-related genes were correlated with mitochondria fusion and fission genes. These results suggest that mitochondria fusion and fission might be a crucial target for irradiation-induced white matter injury.

Mitochondria are dynamic organelles that constantly change shape as a result of a balance between fusion and fission, and modulating the balance of mitochondrial fission–fusion is crucial for maintaining cellular homeostasis [53]. Irradiation results in nuclear DNA damage, and there is evidence suggesting that such nuclear damage occurs secondary to mitochondrial injury [54]. The expression of the mitochondrial fusion and fission-related genes *Opa1* and *Fis1* was searched for in the public database “Mouse Cell Atlas,” which uses single-cell RNA sequencing to determine the cell type composition of major mouse organs and constructs a basic scheme for the Mouse Cell Atlas (http://bis.zju.edu.cn/MCA/) [55], and we found that mitochondrial fusion and fission occur in most cells of central nervous system, including neurons, microglia, astrocytes, and oligodendrocytes. Among the cell types we searched, mitochondrial fusion and fission occurred most frequently in oligodendrocytes. We do not yet have single-cell RNA sequencing data for the mice after irradiation, and more experiments need to be performed on the changes of mitochondrial fusion and fission levels after irradiation in different types of cells in the CNS.

Due to the lack of protection by proteins and histones, mitochondria are very susceptible to irradiation damage [56]. As a result, compensatory mitochondrial fusion after low-dose irradiation is needed to remove the dysfunctional mitochondrial DNA and to maintain respiratory function [57]. At medium or high-dose irradiation, mitochondrial fusion usually decreases [58]. Protein kinase A phosphorylates DRP1 at Ser637 in the conserved GTPase effector domain of rodent animals, and this phosphorylation has been shown to cause a significant decrease in GTPase activity and to inhibit mitochondrial fission [16, 17]. P-DRP1, as a marker of inhibited mitochondrial fission, showed the same tendency as the mitochondria fusion marker OPA1. In this study, their protein levels dramatically decreased in KO mice compared with WT mice under physiological conditions, and the loss of selective autophagy led to decreased mitochondria fusion. Although the differences were significant under physiological conditions, there were no significant changes under physiological conditions in the myelin sheath between KO and WT mice as shown in Fig. 1. Interestingly, the protein levels of P-DRP1 and OPA1 showed no differences in WT mice between the non-irradiated group and the irradiated group, but they were significantly increased in the irradiated KO mice at 5 days after irradiation. We assumed that autophagy deficiency in the KO mice might reduce the sensitivity of mitochondria to irradiation by increasing mitochondrial fusion and inhibiting mitochondrial fission after irradiation, thus providing more energy and molecular precursors for myelin synthesis. These results suggest that autophagy inhibition prevents irradiation-induced subcortical white matter injury by increasing mitochondrial fusion and inhibiting mitochondrial fission. MitoQ is a mitochondria-targeted antioxidant that was reported to reduce irradiation-induced brain injury in mice and to protected mitochondrial respiration after irradiation by altering the level of mitochondrial fusion and fission [58]. This finding suggests that autophagy might be a potential therapeutic target by altering mitochondrial fusion and fission for the treatment of irradiation-induced brain injury.

## Conclusions

In conclusion, selective neural *Atg7* KO reduced irradiation-induced subcortical white matter injury by increasing mitochondrial fusion and inhibiting mitochondrial fission in the juvenile mouse brain. Further studies are needed to clarify the mechanism behind these effects. Our study suggests that inhibition of neural autophagy might be a potential therapeutic target for irradiation-induced brain injury in children being treated for malignant brain tumors.

## Supplementary Information

Below is the link to the electronic supplementary material.

Supplementary file1 (PDF 439 KB)

## Data Availability

All data generated or analyzed during this study are included in the published article.

## Abbreviations

Atg7: Autophagy 7
DEG: Differentially expressed gene
DRP1: Dynamin-related protein 1
FIS1: Mitochondrial fission 1 protein
GFAP: Glial fibrillary acidic protein
Iba-1: Ionized calcium-binding adaptor molecule 1
IL: Interleukin
KC: Keratinocyte-derived chemokine
KO: Knockout
MBP: Myelin basic protein
OPC: Oligodendrocyte progenitor cell
PDGFRα: Platelet derived growth factor receptor α
WT: Wild-type
BBB: Blood–brain barrier

## References

[1] Baron Nelson M, Compton P, Patel SK, Jacob E, Harper R (2013). Central nervous system injury and neurobiobehavioral function in children with brain tumors: a review of the literature. Cancer Nurs.

[2] Zajac-Spychala O, Pawlak MA, Karmelita-Katulska K, Pilarczyk J, Derwich K, Wachowiak J (2017). Long-term brain structural magnetic resonance imaging and cognitive functioning in children treated for acute lymphoblastic leukemia with high-dose methotrexate chemotherapy alone or combined with CNS radiotherapy at reduced total dose to 12 Gy. Neuroradiology.

[3] Rose SR, Horne VE, Howell J, Lawson SA, Rutter MM, Trotman GE, Corathers SD (2016). Late endocrine effects of childhood cancer. Nat Rev Endocrinol.

[4] Paulino AC, Fowler BZ (2005). Secondary neoplasms after radiotherapy for a childhood solid tumor. Pediatr Hematol Oncol.

[5] Anderson NE (2003). Late complications in childhood central nervous system tumour survivors. Curr Opin Neurol.

[6] Xie C, Ginet V, Sun Y, Koike M, Zhou K, Li T, Li H, Li Q (2016). Neuroprotection by selective neuronal deletion of Atg7 in neonatal brain injury. Autophagy.

[7] Ai X, Ye Z, Yao Y, Xiao J, You C, Xu J, Huang X, Zhong J (2020). Endothelial autophagy: an effective target for radiation-induced cerebral capillary damage. Sci Rep.

[8] Hao Y, Li W, Wang H, Zhang J, Yu C, Tan S, Xu X, Dong J (2018). Autophagy mediates the degradation of synaptic vesicles: a potential mechanism of synaptic plasticity injury induced by microwave exposure in rats. Physiol Behav.

[9] Wang Y, Zhou K, Li T, Xu Y, Xie C, Sun Y, Rodriguez J, Zhang S (2019). Selective neural deletion of the atg7 gene reduces irradiation-induced cerebellar white matter injury in the juvenile mouse brain by ameliorating oligodendrocyte progenitor cell loss. Front Cell Neurosci.

[10] Wang Y, Zhou K, Li T, Xu Y, Xie C, Sun Y, Zhang Y, Rodriguez J (2017). Inhibition of autophagy prevents irradiation-induced neural stem and progenitor cell death in the juvenile mouse brain. Cell Death Dis.

[11] Zhang X, Rocha-Ferreira E, Li T, Vontell R, Jabin D, Hua S, Zhou K, Nazmi A (2017). gammadeltaT cells but not alphabetaT cells contribute to sepsis-induced white matter injury and motor abnormalities in mice. J Neuroinflammation.

[12] Li T, Li K, Zhang S, Wang Y, Xu Y, Cronin SJF, Sun Y, Zhang Y (2020). Overexpression of apoptosis inducing factor aggravates hypoxic-ischemic brain injury in neonatal mice. Cell Death Dis.

[13] Yu G, Wang LG, Han Y, He QY (2012). clusterProfiler: an R package for comparing biological themes among gene clusters. OMICS.

[14] Sharma NK, Stone S, Kumar VP, Biswas S, Aghdam SY, Holmes-Hampton GP, Fam CM, Cox GN (2019). Mitochondrial degeneration and autophagy associated with delayed effects of radiation in the mouse brain. Front Aging Neurosci.

[15] Kempf SJ, Moertl S, Sepe S, von Toerne C, Hauck SM, Atkinson MJ, Mastroberardino PG, Tapio S (2015). Low-dose ionizing radiation rapidly affects mitochondrial and synaptic signaling pathways in murine hippocampus and cortex. J Proteome Res.

[16] Knott AB, Perkins G, Schwarzenbacher R, Bossy-Wetzel E (2008). Mitochondrial fragmentation in neurodegeneration. Nat Rev Neurosci.

[17] Chang CR, Blackstone C (2007). Cyclic AMP-dependent protein kinase phosphorylation of Drp1 regulates its GTPase activity and mitochondrial morphology. J Biol Chem.

[18] Shimizu S (2018). Biological roles of alternative autophagy. Mol Cells.

[19] Xiong J (2015). Atg7 in development and disease: panacea or Pandora's Box?. Protein Cell.

[20] Wang HJ, Wei JY, Liu DX, Zhuang SF, Li Y, Liu H, Ban M, Fang WG (2018). Endothelial Atg7 deficiency ameliorates acute cerebral injury induced by ischemia/reperfusion. Front Neurol.

[21] Shi H, Wang J, Huang Z, Yang Z (2018). IL-17A induces autophagy and promotes microglial neuroinflammation through ATG5 and ATG7 in intracerebral hemorrhage. J Neuroimmunol.

[22] Gao C, Yan Y, Chen G, Wang T, Luo C, Zhang M, Chen X, Tao L (2020). Autophagy activation represses pyroptosis through the IL-13 and JAK1/STAT1 pathways in a mouse model of moderate traumatic brain injury. ACS Chem Neurosci.

[23] Menzies FM, Fleming A, Rubinsztein DC (2015). Compromised autophagy and neurodegenerative diseases. Nat Rev Neurosci.

[24] Galluzzi L, Bravo-San Pedro JM, Blomgren K, Kroemer G (2016). Autophagy in acute brain injury. Nat Rev Neurosci.

[25] Balentova S, Adamkov M (2015). Molecular, cellular and functional effects of radiation-induced brain injury: a review. Int J Mol Sci.

[26] Bull C, Cooper C, Lindahl V, Fitting S, Persson AI, Grander R, Alborn AM, Bjork-Eriksson T (2017). Exercise in adulthood after irradiation of the juvenile brain ameliorates long-term depletion of oligodendroglial cells. Radiat Res.

[27] Panagiotakos G, Alshamy G, Chan B, Abrams R, Greenberg E, Saxena A, Bradbury M, Edgar M (2007). Long-term impact of radiation on the stem cell and oligodendrocyte precursors in the brain. PLoS ONE.

[28] Fukuda H, Fukuda A, Zhu C, Korhonen L, Swanpalmer J, Hertzman S, Leist M, Lannering B (2004). Irradiation-induced progenitor cell death in the developing brain is resistant to erythropoietin treatment and caspase inhibition. Cell Death Differ.

[29] Begolly S, Olschowka JA, Love T, Williams JP, O'Banion MK (2018). Fractionation enhances acute oligodendrocyte progenitor cell radiation sensitivity and leads to long term depletion. Glia.

[30] Atkinson SL, Li YQ, Wong CS (2005). Apoptosis and proliferation of oligodendrocyte progenitor cells in the irradiated rodent spinal cord. Int J Radiat Oncol Biol Phys.

[31] Hu X, Leak RK, Shi Y, Suenaga J, Gao Y, Zheng P, Chen J (2015). Microglial and macrophage polarization-new prospects for brain repair. Nat Rev Neurol.

[32] Burda JE, Bernstein AM, Sofroniew MV (2016). Astrocyte roles in traumatic brain injury. Exp Neurol.

[33] Raffaele S, Lombardi M, Verderio C, Fumagalli M (2020). TNF production and release from microglia via extracellular vesicles: impact on brain functions. Cells.

[34] Karve IP, Taylor JM, Crack PJ (2016). The contribution of astrocytes and microglia to traumatic brain injury. Br J Pharmacol.

[35] Dupont N, Jiang S, Pilli M, Ornatowski W, Bhattacharya D, Deretic V (2011). Autophagy-based unconventional secretory pathway for extracellular delivery of IL-1beta. EMBO J.

[36] Piccioli P, Rubartelli A (2013). The secretion of IL-1beta and options for release. Semin Immunol.

[37] Kraya AA, Piao S, Xu X, Zhang G, Herlyn M, Gimotty P, Levine B, Amaravadi RK (2015). Identification of secreted proteins that reflect autophagy dynamics within tumor cells. Autophagy.

[38] Ponpuak M, Mandell MA, Kimura T, Chauhan S, Cleyrat C, Deretic V (2015). Secretory autophagy. Curr Opin Cell Biol.

[39] Cho KS, Lee JH, Cho J, Cha GH, Song GJ (2020). Autophagy modulators and neuroinflammation. Curr Med Chem.

[40] Nakahira K, Haspel JA, Rathinam VA, Lee SJ, Dolinay T, Lam HC, Englert JA, Rabinovitch M (2011). Autophagy proteins regulate innate immune responses by inhibiting the release of mitochondrial DNA mediated by the NALP3 inflammasome. Nat Immunol.

[41] Shi CS, Shenderov K, Huang NN, Kabat J, Abu-Asab M, Fitzgerald KA, Sher A, Kehrl JH (2012). Activation of autophagy by inflammatory signals limits IL-1beta production by targeting ubiquitinated inflammasomes for destruction. Nat Immunol.

[42] Paul S, Kashyap AK, Jia W, He YW, Schaefer BC (2012). Selective autophagy of the adaptor protein Bcl10 modulates T cell receptor activation of NF-kappaB. Immunity.

[43] Harris J, Hartman M, Roche C, Zeng SG, O'Shea A, Sharp FA, Lambe EM, Creagh EM (2011). Autophagy controls IL-1beta secretion by targeting pro-IL-1beta for degradation. J Biol Chem.

[44] Claude-Taupin A, Jia J, Mudd M, Deretic V (2017). Autophagy's secret life: secretion instead of degradation. Essays Biochem.

[45] Han W, Umekawa T, Zhou K, Zhang XM, Ohshima M, Dominguez CA, Harris RA, Zhu C (2016). Cranial irradiation induces transient microglia accumulation, followed by long-lasting inflammation and loss of microglia. Oncotarget.

[46] Sandor N, Walter FR, Bocsik A, Santha P, Schilling-Toth B, Lener V, Varga Z, Kahan Z (2014). Low dose cranial irradiation-induced cerebrovascular damage is reversible in mice. PLoS ONE.

[47] Jacob J, Durand T, Feuvret L, Mazeron JJ, Delattre JY, Hoang-Xuan K, Psimaras D, Douzane H (2018). Cognitive impairment and morphological changes after radiation therapy in brain tumors: a review. Radiother Oncol.

[48] Zhang L, Huang P, Chen H, Tan W, Lu J, Liu W, Wang J, Zhang S (2017). The inhibitory effect of minocycline on radiation-induced neuronal apoptosis via AMPKalpha1 signaling-mediated autophagy. Sci Rep.

[49] Liu Y, Yan J, Sun C, Li G, Li S, Zhang L, Di C, Gan L (2018). Ameliorating mitochondrial dysfunction restores carbon ion-induced cognitive deficits via co-activation of NRF2 and PINK1 signaling pathway. Redox Biol.

[50] Tonnies E, Trushina E (2017). Oxidative stress, synaptic dysfunction, and Alzheimer's disease. J Alzheimers Dis.

[51] Luca M, Luca A (2019). Oxidative stress-related endothelial damage in vascular depression and vascular cognitive impairment: beneficial effects of aerobic physical exercise. Oxid Med Cell Longev.

[52] Gonzalez S, Fernando R, Berthelot J, Perrin-Tricaud C, Sarzi E, Chrast R, Lenaers G, Tricaud N (2015). In vivo time-lapse imaging of mitochondria in healthy and diseased peripheral myelin sheath. Mitochondrion.

[53] Fenton AR, Jongens TA, Holzbaur ELF (2020). Mitochondrial dynamics: shaping and remodeling an organelle network. Curr Opin Cell Biol.

[54] Azzam EI, Jay-Gerin JP, Pain D (2012). Ionizing radiation-induced metabolic oxidative stress and prolonged cell injury. Cancer Lett.

[55] Han X, Wang R, Zhou Y, Fei L, Sun H, Lai S, Saadatpour A, Zhou Z (2018). Mapping the mouse cell atlas by microwell-Seq. Cell.

[56] Kam WW, Banati RB (2013). Effects of ionizing radiation on mitochondria. Free Radic Biol Med.

[57] Chen H, Vermulst M, Wang YE, Chomyn A, Prolla TA, McCaffery JM, Chan DC (2010). Mitochondrial fusion is required for mtDNA stability in skeletal muscle and tolerance of mtDNA mutations. Cell.

[58] Gan L, Wang Z, Si J, Zhou R, Sun C, Liu Y, Ye Y, Zhang Y (2018). Protective effect of mitochondrial-targeted antioxidant MitoQ against iron ion (56)Fe radiation induced brain injury in mice. Toxicol Appl Pharmacol.

