# Supplementary material for: Autophagy Inhibition Reduces Irradiation-Induced Subcortical White Matter Injury Not by Reducing Inflammation, but by Increasing Mitochondrial Fusion and Inhibiting Mitochondrial Fission
Source: Mol Neurobiol. 2021 Dec 28;59(2):1199–213. doi: 10.1007/s12035-021-02653-x (PMC8857090; doi:10.1007/s12035-021-02653-x)
Supplement: Supplementary file 1 — Supplementary file1 (PDF 439 KB) [file 12035_2021_2653_MOESM1_ESM.pdf]

Fig.S1

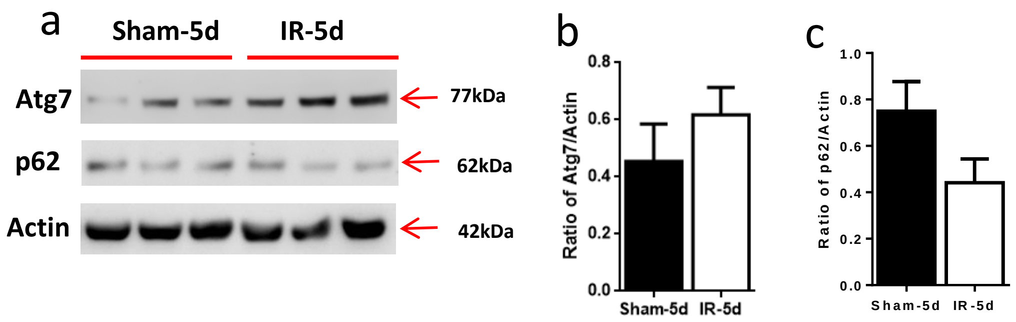

Fig. S1 The influence of irradiation on autophagy in the wildtype mice. **a** Representative immunoblots of Atg7 and p62 in the wildtype mice under physiological conditions and after irradiation. **b-c.** Quantification of Atg7 or p62 at 5 days after irradiation.

Fig.S2

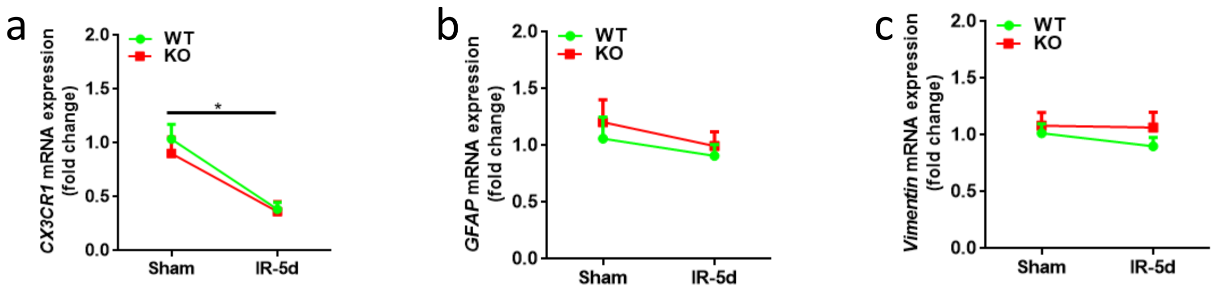

Fig. S2 The expression of microglia and astrocyte-related genes in the subcortical white matter after irradiation. a. Bar graph showing the mRNA expression of *CX3CR1* in the cortical tissue including the subcortical white matter at 5 days after irradiation. **b-c**. Bar graphs showing the mRNA expression of *GFAP* and *Vimentin* at 5 days after irradiation. n = 5/group for qRT-PCR. *$p < 0.05$.

Fig. S3

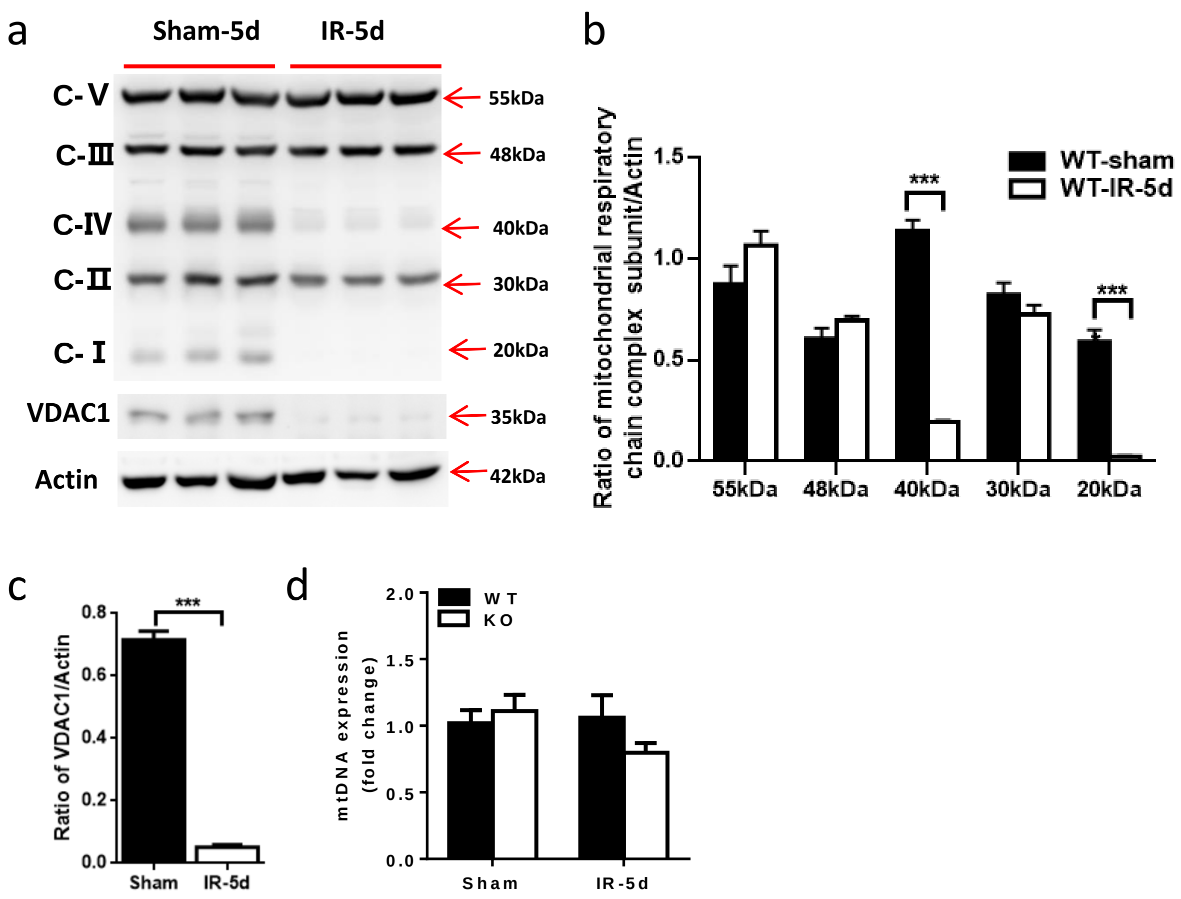

Fig. S3 Mitochondrial biogenesis after irradiation. a. Immunoblotting of individual respiratory chain complexes (C-I, C-II, C-III, C-IV, C-V) subunits and VDAC1 in the wildtype mice after irradiation. **b-c.** Quantification of respiratory chain complex subunits or VDAC1 at 5 days after irradiation. **d**. mtDNA copy number had no changes at 5 days after irradiation. ***$p < 0.001$.

Fig.S4

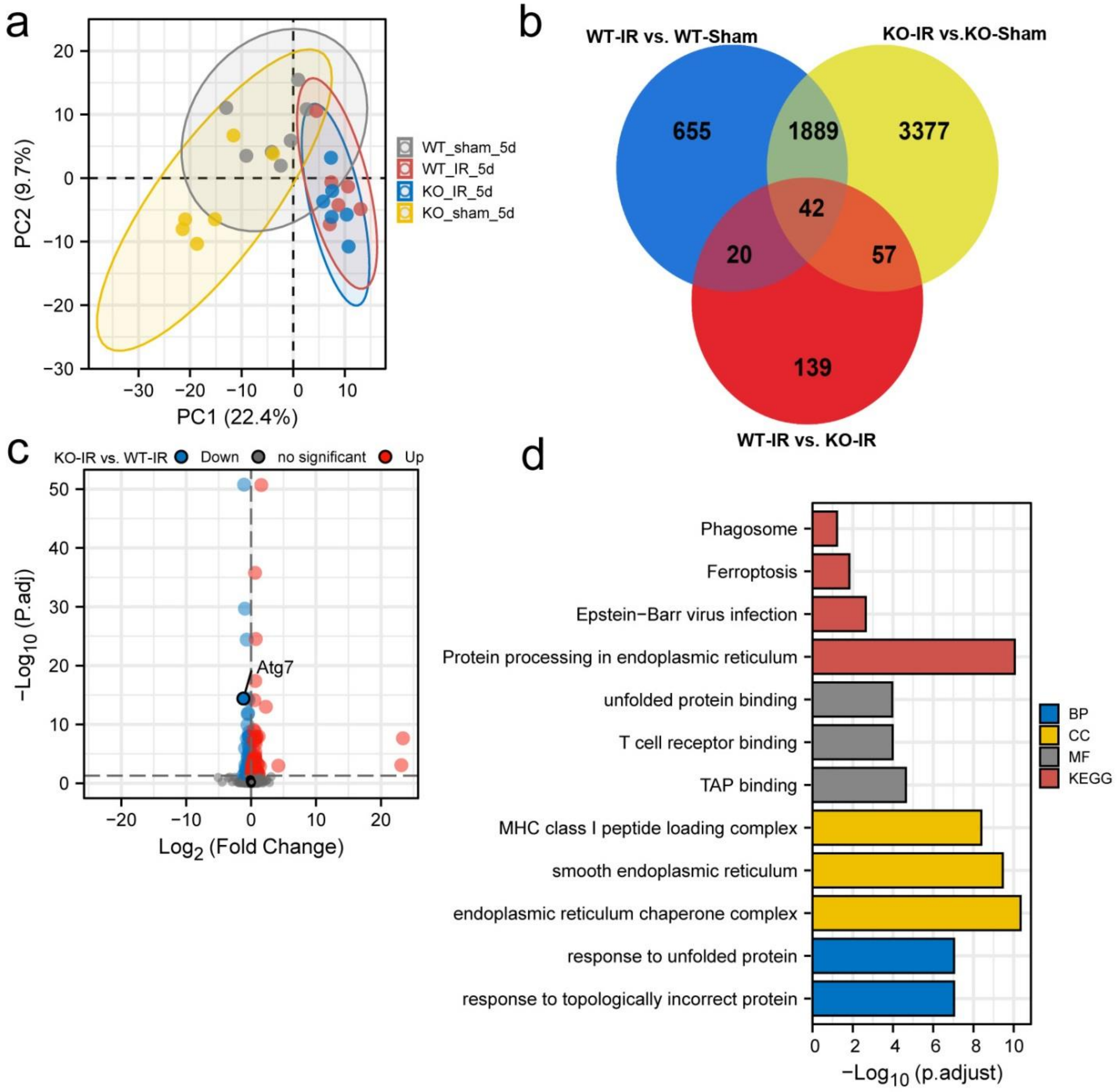

Fig. S4. The transcriptional levels of Atg7-deficient mice are also quite different from WT mice after brain irradiation. a. Principal component analysis (PCA) shows similarities between the Atg7 KO and WT mice after irradiation, and brain irradiation became the main factor leading to the differentiation of the four groups. **b**. A Venn diagram showing the number of DEGs in the three comparisons identified by RNA-Seq. **c**. Volcano plot (p-value versus fold change ratio IR-KO/WT) at 5d after brain irradiation, and significantly overexpressed genes are represented as 'red' dots and significant down-regulated genes are represented as 'blue' dots. **d**. The functions of the differentially expressed genes between Atg7 KO and WT mice after irradiation were predicted using GO and KEGG analyses.
